# Translational medicine research on early detection and early diagnosis of colorectal cancer in China

**DOI:** 10.1186/1479-5876-10-S2-A13

**Published:** 2012-10-17

**Authors:** Suzhan Zhang, Yanqin Huang, Shanrong Cai, Jiekai Yu, Wenhong Xu, Wen Meng, Shu Zheng

**Affiliations:** 1 Cancer Institute (Key Laboratory of Cancer Prevention and Intervention, China National Ministry of Education, Key Laboratory of Molecular Biology in Medical Sciences, Zhejiang Province, China), The Second Affiliated Hospital, Zhejiang University School of Medicine, Hangzhou, Zhejiang 310009, China

## Background

Colorectal cancer (CRC) is the most common cancer in China. The incidence of CRC is increasing rapidly. The early detection and early diagnosis is the effective way to decreasing the mortality and increasing the survival rate.

## Materials and methods

From 2007, a CRC screening program was begun in three cities (Hangzhou, Haerbing, and Shanghai city, aimed at finding more early CRC cases and premalignant lesions such as adenoma. The two steps screening model was applied. As the primary screening, the Simultaneous iFOBT (immunochemical Fecal Occult Blood Testing) and high risk factors questionnaire investigation (HRFQ) will be employed for selection the high risk population from target population. Then the high risk population will be further selected by colonoscopy.

## Results

the compliance of the two steps screening model was only 34.88% (1650/ 4730) in city target. The overall positive rate in the first screening stage was 13.5% (4,730 of 35,037). In the second stage, the positive rate of total colorectal neoplasm was 27.27% (450/1650) in the total study population. The detected ratesof cancer, adenoma, non-adenomatous polyps, and advanced neoplasm were 13%.

## Conclusions

The combining iFOBT and HRFQ as primary screening methods is an efficient CRC screening strategy in economically and medically underserved population. But the novel early CRC markers were still needed to improve the compliance of the screening program.

